# Misattribution of Dyspnea: Unveiling Atrial Myxoma as the True Culprit

**DOI:** 10.7759/cureus.58790

**Published:** 2024-04-22

**Authors:** Ali M Al Zweihary

**Affiliations:** 1 Department of Medicine, Unaizah College of Medicine and Medical Sciences, Qassim University, Unaizah, SAU

**Keywords:** tumor, cardiac surgery, echocardiography, dyspnea, myxoma

## Abstract

Cardiac myxomas are the most common primary tumors of cardiac neoplasm, predominantly originating within the left atrium (LA). In the present case, a 41-year-old male, identified as a heavy smoker for 15 years, previously diagnosed with chronic obstructive pulmonary disease (COPD) and currently undergoing treatment, presented with a history of dyspnea persisting for one year. Initially, the patient presented to the internal medicine outpatient clinic and was diagnosed with an exacerbation of COPD, but subsequent evaluation revealed the presence of a large mobile pedunculated mass situated in the LA using echocardiography. Subsequently, the mass was surgically excised using a median sternotomy approach. The histopathological examination confirmed cardiac myxoma. This occurrence underscores the significance of considering cardiac myxoma as a plausible differential diagnosis in instances of dyspnea to avert potential complications.

## Introduction

Atrial myxomas are the most common primary masses among heart tumors. They possess a health concern considering how they can be confused with and exhibit a wide range of clinical features of many cardiovascular and systemic diseases. These benign cardiac tumors are frequently found in the left atrium (LA) and produce diverse symptoms that can be related to the tumor size, tumor position, and the presence of any obstruction of the normal function of the heart. Even though these are benign tumors, they might cause serious consequences, including embolization, interruption of blood flow, and then, subsequently, heart failure [1]. This means that early detection and treatment, being the main approach, are essential for tackling this issue. The clinical presentation of atrial myxomas has been observed to exhibit varying degrees of signs and symptoms that range from aseptically with no symptoms to the ones whose manifestations are severe and life-threatening. Common complaints are that, during positional changes, a patient experiences dyspnea, and thus dizziness, fainting attack, and embolic events occur systemically [2]. The formless kind of symptoms have made diagnosis challenging, so physicians should maintain a high level of suspicion and have a comprehensive evaluation procedure. In this situation, however, echocardiography, in particular, plays a leading role in other aspects, such as detecting and defining these intra-cardiac masses. 

## Case presentation

**Figure 1:**
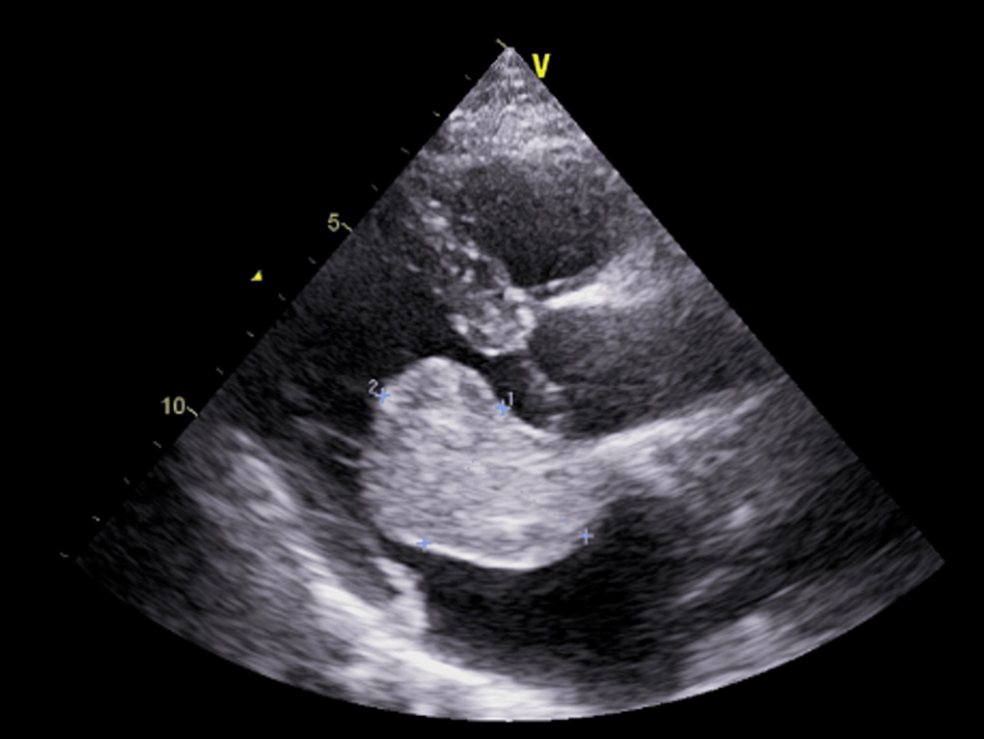
Echocardiography showing a 3.7 x 5.1 cm tumor in the left atrium protruding in the left ventricle

A 41-year-old male who has been smoking for over 15 years was previously diagnosed with chronic obstructive pulmonary disease (COPD) and has been on bronchodilators. The patient was seeing a clinician in the outpatient clinic because he experienced shortness of breath that started almost one year ago. The breathlessness has worsened, specifically in the upright position, and it has been improved marginally by utilizing a beta agonist and adopting a supine position. After that, the patient was diagnosed as a case of COPD exacerbation at that moment, accompanied by optimization of his prescribed medications. Following that, after six weeks, the patient returns with deteriorating symptoms, showing no response to the prescribed drugs. These clinical features, positional and progressive worsening dyspnea in a short period despite the optimization of prescribed medications, promoted in-depth clinical investigations. Further examination done with the patient showed no sign of any acute distress; nevertheless, he showed signs of discomfort when put in an upright position. Vital signs were within normal limits, and the cardiac examination showed no particular abnormalities except for apically located mid to late diastolic murmur that disappeared in a recumbent position. An electrocardiogram (ECG) and standard laboratory panel, including the complete blood count, electrolytes, renal function tests, liver function tests, and thyroid function, were done, and those tests showed all values within normal limits. Lastly, transthoracic echocardiography showed a mobile mass of 51 x 37 mm seen in the LA being attached to the interatrial septum, which is prolapsing through the mitral valve into the left ventricle during diastole, consistent with LA myxoma (Figure 1).

**Figure 2:**
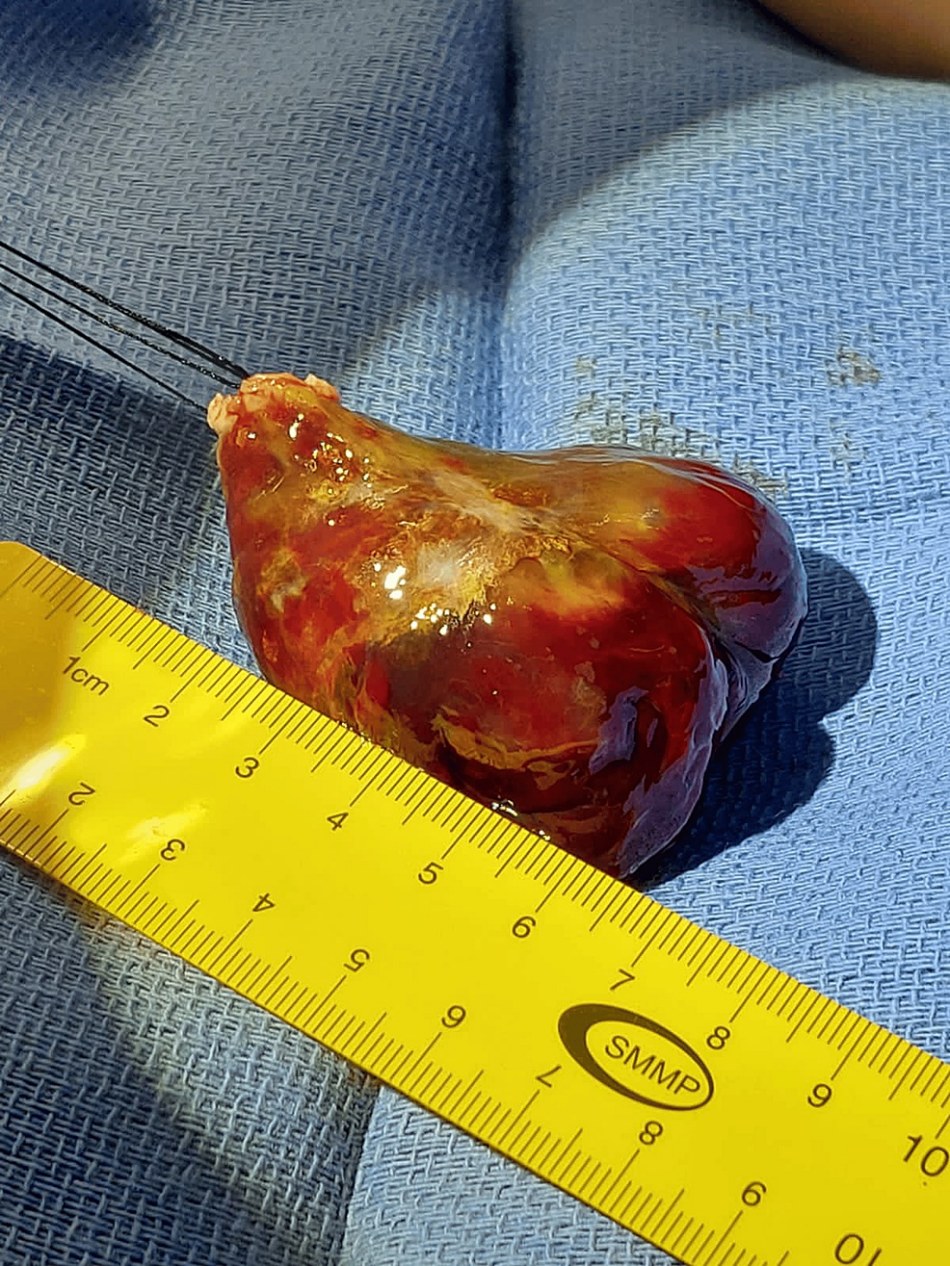
Cardiac myxoma removed from the left atrium

Transesophageal echocardiography (TEE) was used later to line out the tumor's extent, informing the effect of a myxoma on the LA mechanic as a way to clarify the diagnosis. The significant diagnosis revealed that the patient needed an immediate referral to cardiothoracic surgery. The surgeons took up the median sternotomy approach. The tumor was carefully disaggregated with the entirety of it removed (Figure 2) and the histopathological examination confirmed its presence as a local tumor, indicated by its gelatinous structure and myxoid stroma laden with dispersed cells.

The next morning, the patient experienced no shortness of breath. Moreover, the patient has been periodically followed up in the outpatient clinic for one year. The patient stated that his dyspnea had disappeared completely, and echocardiography showed no recurrence of myxoma. The storyline reflects critically essential elements of atrial myxomas. Initially, it highlights how atrial myxoma patients, to whom the above symptoms are attributed, should always be considered for having the condition even despite a definite background.

Therefore, the case highlights the immediate surgical management, which not only improves the symptom presentation but also prevents devastating consequences, such as embolization, heart failure, or sudden cardiac death. 

## Discussion

The case supports the diagnostic challenges of clinicians facing patients with dyspnea which includes respiratory and cardiac sources and how confounding factors may delay the diagnosis of clinically relevant conditions, such as atrial myxomas. This clinical entity also postulates the importance of imaging modalities and the effectiveness of surgical intervention in managing this condition. Atrial myxomas have a low prevalence, with an estimated incidence rate of 0.5 per million people yearly [3]. The latter, paired with the possibility of patients presenting multiple undifferentiated symptoms such as syncope, dyspnea, or positional breathlessness, explains the necessity for a high suspicion index in the cases of unsolved conditions that manifest as syncope, unsupported by existing medical information [4]. The intracardiac tumor not unveiling itself on a systematic physical examination and repeated laboratory analyses is a case in point that the classical diagnostic (non-invasive) techniques are inadequate in recognizing intracardiac masses [5]. This makes the vital contribution of echocardiography (transthoracic and transesophageal) clearer as it is always used to visualize and characterize such tumors.

Atrial myxomas have the characteristic feature of presenting themselves the way heterogeneity signs, which range from asymptomatic to accidental discovery to severe, life-threatening conditions. These include surgical obstruction, embolization, systemic manifestations, and stroke [6]. Embolization of myxomas placed in the LA is the most dangerous for systemic embolization in the brain, kidneys, or peripheral area. Also, the cardiac position at which the tumors arise can facilitate obstruction of the bloodstream, which may resemble mitral valve stenosis, and that can lead to congestive heart failure or sudden cardiac death if not attended promptly [7]. Most atrial myxomas are sporadic, just a few percent are related to familial syndromes like the Carney complex, whose main symptoms are inclined to several tumor formations [8]. Given this genetic relationship, physicians must have adequate information about the patient's atrial myxomas family history and, in some cases, help patients with a diagnosis get genetic counseling and testing, especially those who are young or the ones with multiple or recurrent tumors.

A pathway that is clearly in favor of surgical intervention is the therapeutic management of an atrial myxoma, with complete removal of the tumor being the main goal [9]. Regarding the surgical intervention, the patient has just gone on symptomatic and electrocardiographic resolution and returned to normal cardiac functioning, indicating that the approach is effective. Nevertheless, the chance of relapse, probably low, is very important to the medical personnel as they continue to monitor periodically for signs of returned tumors. In these cases, however, the recurrence rate is elevated as a family history of myxoma or incomplete excision increases the reoccurrence probability [10]. The approach underlines the benefits of transdisciplinary improvement, including cardiologists, cardiothoracic surgeons, radiologists, and pathologists in a multimodal approach to this illness. It also clarifies that good and consistent patient education is important based on the nature of the condition and the reason behind the surgeries.

## Conclusions

Cardiac myxomas are an important clinical entity of cardiac tumors. This is mostly because they appear almost twice as much as the primary heart tumors. These identifiable lesions, more often than not, need a tedious diagnostic process before confirming their resemblance to the broad range of pathologies that often present with cardiovascular symptoms and disease. Echocardiography emerges as a main diagnostic modality offering crucial insights into tumor characteristics that guide management strategies. Surgical removal is the gold standard approach to the treatment, affording to both show symptom disappearance and the elimination of life-threatening complications, including embolic events and sudden cardiac death. Therefore, timely diagnosis and surgical intervention at the earliest possible moment are mandatory in managing cardiac myxomas, which represent an example of the particular case balance among early diagnosis and immediate medical intervention to reduce complication severity and prolong patient life and wellness.
